# The Effect of Honey Powder Addition on Chosen Quality Properties of Model Chicken Products

**DOI:** 10.3390/foods13244163

**Published:** 2024-12-22

**Authors:** Radosław Bogusz, Anna Onopiuk, Marta Chmiel, Anna Piotrowska, Eliza Kostyra, Edyta Lipińska, Joanna Bryś, Katarzyna Samborska, Dorota Pietrzak

**Affiliations:** 1 Department of Food Engineering and Process Management, Institute of Food Sciences, Warsaw University of Life Sciences—SGGW, 159c Nowoursynowska Street, 02-776 Warsaw, Poland; katarzyna_samborska@sggw.edu.pl; 2 Department of Technique and Food Development, Institute of Human Nutrition Sciences, Warsaw University of Life Sciences—SGGW, 159c Nowoursynowska Street, 02-776 Warsaw, Poland; anna_onopiuk@sggw.edu.pl; 3 Department of Food Technology and Assessment, Institute of Food Sciences, Warsaw University of Life Sciences—SGGW, 159c Nowoursynowska Street, 02-776 Warsaw, Poland; dorota_pietrzak@sggw.edu.pl; 4 Department of Functional and Organic Food, Institute of Human Nutrition Sciences, Warsaw University of Life Sciences—SGGW, 159c Nowoursynowska Street, 02-776 Warsaw, Poland; anna_piotrowska@sggw.edu.pl (A.P.); eliza_kostyra@sggw.edu.pl (E.K.); 5 Department of Food Biotechnology and Microbiology, Institute of Food Sciences, Warsaw University of Life Sciences—SGGW, 159c Nowoursynowska Street, 02-776 Warsaw, Poland; edyta_lipinska@sggw.edu.pl; 6 Department of Chemistry, Institute of Food Sciences, Warsaw University of Life Sciences—SGGW, 159c Nowoursynowska Street, 02-776 Warsaw, Poland; joanna_brys@sggw.edu.pl

**Keywords:** natural antioxidants, chicken meat, lipid oxidation, microbiological quality, sensory properties, volatile compounds profile

## Abstract

The objective of our paper was to evaluate the effect of honey powder addition on the quality of model chicken products over 14 days of refrigerated storage. Three model chicken product variants were produced: C—control, HP1%, HP2%—with 1 or 2% of honey powder addition. The cooking loss, basic chemical composition, water activity, texture, color, lipid oxidation (TBARS and PDSC), microbiological and sensory quality, and volatile compounds profile were determined. The adverse changes in lipids were slower in products with honey powder added compared to control product, revealing lower TBARS index values and longer oxidation induction times. After 14 days of storage, HP2% products showed significantly lower (up to 50%) TBARS values than control products. Furthermore, honey powder addition reduced the growth of psychrotrophic and lactic acid bacteria for up to 14 days of storage in comparison to the control products. However, deterioration of the volatile compounds profile (presence of alcohols and sulfur compounds) and occurrence of storage odor and flavor had an impact on the poorer sensory desirability of the control and HP1% products. Additional research is necessary aiming to improve the sensory quality of products with honey powder addition.

## 1. Introduction

Consumers, when purchasing convenience food, are increasingly paying attention to its quality. They want it to be tasty and healthy, with as few synthetic additives as possible and, at the same time, a long shelf-life [1,2]. Very popular and willingly purchased among consumers is poultry meat. It is due to its appealing sensory properties (deliciousness, tenderness, and juiciness), ease of preparation and versatility in culinary use, and low price. Moreover, it is a valuable source of complete proteins, vitamins, and some minerals and is characterized by a low fat content. From the producers’ view, it is a good raw material for the production of convenience food [3,4]; however, such meat with high polyunsaturated fatty acids (PUFAs) content is prone to lipid oxidation [5,6]. Poultry meat is also more susceptible to the growth of undesirable microflora (including *Salmonella* sp. and *Campylobacter jejuni*) compared to meat from large slaughter animals [5,7].

Lipid oxidation can adversely impact the sensory attributes of products made from poultry meat, cause the formation of toxic compounds, and reduce their nutritional value. For this purpose, antioxidants are used. Synthetic antioxidants are mostly applied in the food industry [8,9]. Recently, the meat industry has been striving to generate new and attractive products with fewer synthetic additives. Therefore, alternative antioxidants of natural origin are being sought that will have comparable technological properties and may be employed in producing meat products [1,9]. These include a variety of herbs and spices, including oregano, rosemary, thyme, ginger, turmeric, peppers, red chili, or hemp seed pomace [1,3,7,10], as well as extracts such as sage, lavender, thyme, oregano, marjoram, lovage, buckwheat hulls, Baikal skullcap root, and propolis [7,11,12,13,14]. Attempts have also been made to use honey to reduce adverse changes in lipids in poultry meat [15,16], but these have not been widely studied.

Honey is consumed worldwide for its distinctive taste and aroma, while food manufacturers try to add it during the production process to obtain new innovative products. Honey improves the flavor of meat and diverse spices, helps in the binding of components, and may improve the texture of products. Additionally, honey addition may mask the saltiness of meat products and add color as it promotes the caramelization of sugars [16]. And as it is known, the darker crust color of roasted or fried products is more willingly chosen by consumers than products obtained by other cooking methods [17,18]. Nonetheless, the unfavorable properties of honey, such as high density and viscosity, lead to challenges in its transportation, storage, or dosage [19,20]. Therefore, honey in powder form could crucially alleviate these problems and also help open up new opportunities for producers to use this form of honey.

The honey powder has many benefits, including longer shelf-life, ease of dosing, storage, and transport, and the potential to blend with other diverse dry food ingredients. Several examples of applications of honey powder in food production have been reported in the literature, including its addition to bread formulations to improve dough rheology, sensory, and textural properties [21]; its use as a sucrose substitute in bakery goods [22]; and in the production of cookies as a sugar substitute to improve the chemical and nutritional properties [23]. Since there are still many unanswered questions about the influence of the addition of honey powder on the quality properties of meat products, it was decided to dedicate this work to clarifying these questions.

New meat products from the convenience food sector, such as products containing honey powder, seem to be an appealing option for consumers interested in consuming foods with beneficial ingredients. Therefore, this paper aimed to investigate the effect of honey powder addition on the quality characteristics of model chicken products stored under refrigerated conditions. The cooking loss, basic chemical composition, water activity, shear force, color, lipid oxidation (thiobarbituric acid reactive substances—TBARS and Pressure Differential Scanning Calorimetry—PDSC), microbiological quality, sensory quality, and volatile compounds profile have been evaluated.

## 2. Materials and Methods

### 2.1. Materials

Buckwheat honey was obtained from a Beekeeping Farm ‘Polski Nektar’ (Siennica, Mińsk Mazowiecki, Poland). The study employed Nutriose FM06 (Roquette, Lestrem, France), a carrier material recognized for its prebiotic properties. Chicken thigh meat, originating from three production batches, was purchased from a local producer.

### 2.2. Honey Powder Production

Liquid feed prior to spray drying was prepared by mechanical mixing of honey and nutriose with distilled water at 20 ± 2 °C. The solids content in the feed solution was 50% (*w/w*), while the ratio of honey solids to carrier solids in the feed (and in the produced powder) was 70:30. The feed solution composition was selected in preliminary experiments.

The prepared liquid feed was dried using NIRO MOBILE MINOR™ pilot-scale spray dryer (GEA, Skanderborg, Denmark) coupled with an external air dehumidification system which consisted of the cooling unit TAEevo TECH020 (MTA, Codogno, Italy) and condensation-adsorption unit ML270 (MUNTERS, Västra Frölunda, Sweden). The liquid feed was dispensed at a rate of 0.22 mL/s and atomized using a rotating disc at a speed of 26,000 rpm (compressed air pressure 4.5 bar). During spray drying, the inlet/outlet air temperature was 80 °C/45 °C [24]. The honey powder was spray-dried once and, after drying, was packed in plastic packages (BOPA/PE, 55 μm) and stored out of light at a temperature of 4 ± 1 °C.

### 2.3. Model Chicken Product Production

The basic recipe composition of meat batters for the model chicken products was as follows: 85% chicken thigh meat (moisture 71.9%, protein 18.6%, and fat 9.5%), 5% egg mass, and 10% hydrated wheat bun (1:1, *w*/*w*). The salt (1.5%) and black pepper (0.1%; Kamis, Poland) were also added in relation to the weight of the meat batter. Three variants of meat batter were prepared: control—without honey powder addition (C) and with honey powder addition in amounts of 1 and 2% (HP1% and HP2%, respectively), in relation to the weight of the meat batter. The amounts of added honey powder that did not impair the product’s sensory characteristics were determined in preliminary studies.

The chicken thighs were minced in a laboratory grinder (Mesko WN40, Mesko-AGD, Skarżysko-Kamienna, Poland) using a mesh size of 4.5 mm. All ingredients for appropriate variants were then mixed for 5 min by using a laboratory robot (Kenwood Major KM 800, Kenwood Ltd., Havant, England, mixer type K). The meat batters were formed using silicone molds (10 × 3.5 × 2 cm), and each meat batter weighed 60 ± 0.5 g. Then, the meat batters were roasted in a convection-steam oven (Rational Self Cooking Center, Rational, Rolling Meadows, IL, USA) at 180 °C (hot air circulation switched on and humidification switched off) until a temperature of 80 °C in the geometric center was reached. After cooling down to ambient temperature and being taken out of the molds, products were placed into multilayer bags PE/PA (75 µm of thickness) and packed using a Multivac C200 vacuum packaging machine (Multivac Sepp Haggenmüller GmbH & Co. KG, Wolfertschwenden, Germany), and kept out of the light for 0, 7, and 14 days under refrigerated conditions (4 ± 2 °C). Three independent production series of each model chicken product were made.

### 2.4. Analyses of Model Chicken Products

#### 2.4.1. Cooking Loss

The cooking loss of model chicken products was determined using a weight method. The weight of meat batters (before roasting) and products (after roasting) were determined, and the difference between them was calculated in relation to the weight of the product before roasting and expressed as a percentage (%).

#### 2.4.2. Basic Chemical Composition

The basic chemical composition of model chicken products was determined following the PN-A-82109:2010 [25] by using the near-infrared reflectance transmission (NIT) spectrometry with artificial neural network (ANN) calibration. The measurement was performed using a FoodScan™ 2 Meat Analyser (FOSS Analytical, Hillerød, Denmark) in 2 repetitions for each product variant in each of the 3 different production series.

#### 2.4.3. Water Activity

The water activity of products was determined by using an Aqua Lab 4TE meter (Decagon Devices, Inc., Pullman, WA, USA) at 24 ± 1 °C and in 2 repetitions for each product variant in each of the 3 different production series.

#### 2.4.4. Shear Force

The shear force of chicken products was determined using a universal testing machine Zwicki 1120 (ZwickRoell GmbH & Co. KG, Ulm, Germany). Before analysis, products were conditioned at 22 ± 1 °C for 1.5 h. The shear force was measured by using a Warner–Bratzler adapter with a V-blade knife. The speed of movement of the measuring head was set at 50 mm/min. The measurement was performed in 4 repetitions for each product variant in each of the 3 different production series.

#### 2.4.5. Color

The color measurement on a cross-section of chicken products was determined using a Minolta^®^ CR-400 colorimeter (Konica–Minolta Inc., Osaka, Japan) and CIE L*a*b* color scale in 5 repetitions for each product in each of 3 different production series. The following settings of the device were used: D65 standard light source, 2° observer, and 8 mm of a measuring head hole. Total color difference (ΔE) between the products without (control) and products with added honey powder (for each storage time individually) was calculated as follows [12]:

(1) $$∆E=\sqrt{{\left({∆L}^{*}\right)}^{2}+{\left({∆a}^{*}\right)}^{2}+{\left({∆b}^{*}\right)}^{2}}$$

where ΔL*, Δa*, and Δb* means the differences between the color components of the control product and the products with added honey powder.

#### 2.4.6. TBARS

The measurement of the thiobarbituric acid reactive substances (TBARS) values of products was determined via the spectrophotometric method [8]. About 5 g of the products were flooded with cold trichloroacetic acid (TCA) solution, and then the antioxidant solution (water/ethanol = 1:1, *v*/*v*), including 0.5% propyl gallate and 0.5% EDTA, was added. The mixture was homogenized, filtered, and transferred to the new test tube. Next, 5 mL of 0.02 M thiobarbituric acid (TBA) solution in a volume of 5 mL was added and subjected to incubation using a water bath (WNB 7 Memmert, Germany) with boiling water for 40 min. The absorbance of the mixtures was taken by using a multimode microplate reader Spark 10M (SparkTM 10M, Tecan Group, Männedorf, Switzerland) at 532 nm against a blank prepared with trichloroacetic acid/water (1:1, *v*/*v*). The TBARS value was measured in 2 repetitions for each chicken product variant in each of the 3 different production series and expressed as a milligram of malondialdehyde per kilogram of the product (mg MDA/kg of the product).

#### 2.4.7. Oxidation Induction Time

Before analysis, fat from products was extracted based on Folch’s method [26]. The oxidation induction time (OIT) of fat extracted from products was determined via a Pressure Differential Scanning Calorimetry (PDSC) method and a DSC Q20 calorimeter (TA Instruments, New Castle, DE, USA) equipped with a high-pressure measuring cell. The experiment was conducted at 120 °C with an initial oxygen pressure of 1350–1400 kPa [26] in 2 repetitions for each product variant in each of the 3 different production series.

#### 2.4.8. Microbiological Quality

The total mesophilic count—TMC (30 °C for 48 h) and the number of psychrotrophic bacteria (4 °C for 10 days) on PCA medium (BTL, Łódź, Poland), lactic acid bacteria—LAB (30 °C for 72 h) on MRS medium (Bio-Rad Laboratories, Inc., Hercules, CA, USA), and Enterobacteriaceae family bacteria (37 °C for 24 h) on Endo medium (BTL, Łódź, Poland) were determined. Samples of products (10 g) were mixed with 90 mL of 1.5% sterile peptone water and homogenized by using a Lab Blender Stomacher 400 Circulator (Seward Ltd., London, UK) for 2 min. A series of ten-fold dilutions from the initial dilution of samples was made, and then 1 mL aliquots from each dilution were dispensed into Petri dishes, to which 15 mL of medium was added. After incubation, colonies were counted and expressed as log CFU/g of product.

#### 2.4.9. Sensory Profiling

The sensory properties of the products were determined using the Quantitative Descriptive Analysis (QDA) method. Thirty features were selected and defined according to the QDA procedure [27]. The final list of attributes included color, eleven odor attributes (meaty, bouillon, roast, fatty, vegetable, sweet, acidic, sterilization, storage, sharp, overall intensity), thirteen flavor and taste cues (meaty, bouillon, roast, fatty, spicy, vegetable, sweet, sour, salty, bitter, pungency, sterilization, storage), four texture descriptors (moisture, hardness, density, particle sensibility), and overall sensory quality. The intensity of the attributes was assessed on a linear unstructured scale of 0–10 cm converted to numerical values (0–10 conventional units, c.u.) anchored at extremes from ’none‘ to ’very intensive‘ for odor, flavor, and taste descriptors. The impressions of texture features and overall sensory quality were evaluated on a scale with boundary terms ‘low’ to ‘high’. In the case of color, anchors on the edges were from ‘light’ to ‘dark’. Overall sensory quality of the products was defined as the impression of the harmony of all examined attributes, with no or only slight intensity of negative cues. Product sensory profiling was carried out directly after production and on day 7 of storage. On day 14 of storage, only orthonasal perception of the products was performed due to the negative changes in odor.

Individual samples of chicken products were presented to the panelists in the amount of 15 g at room temperature (21 ± 2 °C) in plastic beakers coded with 3-digit random numbers and covered with lids. Mineral water was used as a taste neutralizer between the evaluated samples. The samples of products were presented for evaluation following a sequential monadic test.

The sensory profiling of products was performed by nine trained panelists [28] with over 15 years of experience in sensory evaluations of different food products. For statistical data handling, 18 individual results (for each trained panelist, two results from independent sessions) were used. All sensory assessments were performed in the sensory laboratory, fulfilling the general requirements of ISO standard [29]. The individual testing booths were equipped with a computerized system for data acquisition. The sensory tests have been carried out in the morning and early afternoon, with two daily sessions.

#### 2.4.10. Volatile Compound Profile

The Heracles electronic nose e-nose (Alpha M.O.S., Toulouse, France) based on ultrafast gas chromatography (GC) was used to assess products’ volatile organic compounds (VOCs). The instrument is equipped with an autosampler and detector system as well as a data acquisition and processing system. The autosampler was used for automated sampling and injection of the gas sample. The detector system consisted of two chromatographic columns of various polarities: non-polar MXT-5 (10 m × 18 µm, Restek, Centre County, PA, USA) and slightly polar MXT-1701 (10 m × 18 µm, Restek, Centre County, PA, USA) which was connected to two flame ionization detectors (FIDs).

The products were chopped, and a 2 g product was placed in 20 mL headspace vials and capped with a teflon-faced silicon rubber cap. The vials were put in the autosampler and then incubated at 60 °C for 5 min under agitation (500 rpm). After incubation, the headspace was collected into a syringe and injected into GC with a volume of 5000 µL and a speed of 125 µL/s. Hydrogen was used as the carrier gas with a constant flow rate maintained at 250 µL/s level. The injector temperature was 200 °C, and the temperature of the two flame ionization detectors was 260 °C. The injection on the e-nose was performed in 3 replicates. The method was calibrated with an alkane solution (n-butane to n-hexadecane) to convert retention time in Kovats indices and to identify the VOCs using the AroChemBase v 7.0 database (Alpha M.O.S., Tuluse, France).

### 2.5. Statistical Analysis

The one-way analysis of variance (ANOVA) and Tukey’s Honestly Significant Difference (HSD) test were employed to indicate the significant differences in quality parameters between products at different storage times. The analyses were conducted using STATISTICA ver. 13.1 (StatSoft Inc., Tulsa, OK, USA) at a significant level of α = 0.05.

The Principal Component Analysis (PCA) was used to indicate the similarities and differences in the sensory properties and the volatile compounds profile detected in model chicken products. The analyses were conducted using XLSTATS (ver. 2021, Addinsoft, Paris, France).

## 3. Results and Discussion

### 3.1. Cooking Loss and Chemical Composition of Model Chicken Products

Cooking loss provides direct information on the lowering in the weight of meat products due to water loss together with various components during cooking, and it informs about the binding capacity of proteins via water and lipids [30]. The cooking loss of the tested products was comparable (Table 1) and was slightly lower than typical for this type of product [1,3]. The cooking loss between control (C) and HP1% and HP2% model chicken products did not differ significantly (*p* > 0.05). However, products with the addition of honey powder were characterized by only slightly lower cooking loss compared to the control product, probably due to the honey powder’s ability to bind and hold water due to high sugar content [31]. It is related to the links between the hydrogen present in water molecules and the hydroxyl groups available in the amorphous regions of the substrate [32]. Similarly, Turhan et al. [33] demonstrated that adding bee pollen to beef meatballs decreased cooking loss during thermal treatment, probably due to the physical and chemical characteristics of pollen, such as the water-holding ability. A significant decrease in cooking loss was observed in relation to the increased amount of pollen used.

The results of the basic chemical composition of products are shown in Table 1. It was found that products with the addition of honey powder (HP1% and HP2%) exhibited a significantly (*p* ≤ 0.05) lower content of water compared to the control product. Moreover, the HP2% product contained significantly (*p* ≤ 0.05) less protein and more carbohydrates than the control product. The observed differences in carbohydrate content in product variants were due to the addition of honey powder, as honey is primarily composed of sugars [22]. In general, meat products’ chemical composition mainly relies on the composition of the raw materials and additives used. Additives are often used to increase the product’s water content while reducing the fat content, but they also influence the content of other ingredients. These include protein preparations [34,35] or fiber preparations [36,37]. This is confirmed in our study as honey powder—a source of sugars and partially fiber—leads to a significant (*p* ≤ 0.05) increase in carbohydrate content relative to the amount of additive used (Table 1).

### 3.2. Water Activity of Model Chicken Products

Water activity is a significant parameter in assessing a food’s water state. It plays a key role in determining food products’ chemical, biochemical, and microbiological stability and physical properties [7]. The water activity results determined in model chicken products were higher than 0.9 (Table 2) but, simultaneously, consistent with the data from the literature for this type of meat product [7,38].

High water activity generally indicates that products are characterized by a high proportion of free water required for microbial growth. Therefore, such values do not provide these products sufficient microbial stability during storage, which can only be attained below 0.6 [7]. In addition, no data about the effect of adding honey powder on water activity in meat products were found in the accessible literature. Nevertheless, it can be assumed that the water present in the honey powder was bound in its structure and not released to the meat products’ matrix, providing conditions for microbial growth. However, this theory requires further research, e.g., using NMR techniques.

### 3.3. Shear Force of Model Chicken Products

Meat products’ chemical composition and, to a large extent, the additives used in their production, influence their texture. The results of the shear force of the model chicken products are given in Table 3. The addition of honey powder did not significantly (*p* > 0.05) affect the texture of the products. During storage, a significant (*p* ≤ 0.05) increase in shear force values was noted only for the control (C) product [33].

By forming protein–carbohydrate complexes via Maillard reaction [39,40], the shear force of the model chicken products tested can be influenced. Protein–carbohydrate complexes can behave like a gel structure and even form one due to the macromolecular characteristics of proteins and the hydrophilic characteristics of carbohydrates [40,41]. These complexes have the ability to bind and hold water in their structure. The honey powder in the meat product recipe promoted Maillard-type glycosylation, leading to protein molecule aggregation [39]. Consequently, over-aggregation can lead to a deterioration of the texture of gel networks during thermal processing and further storage [39]. In addition, the amount of water bound by the protein–carbohydrate complexes formed may not change. In contrast, the control products may have been further bound during storage, increasing the hardness of the products after 7 and 14 days of storage. Similarly, Turhan et al. [33] demonstrated that bee pollen used in the preparation of beef meatballs decreased their hardness in proportion to the amount of additive used. It was probably due to the physical and chemical characteristics of pollen, such as the water-holding ability.

### 3.4. Color of Model Chicken Products

**Figure 1:**
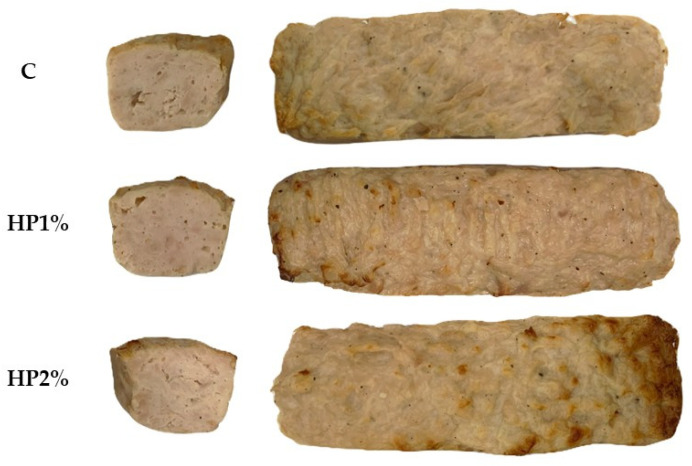
Photos of the cross-section and the surface of the model chicken products after heat treatment. C—control model chicken product—without honey powder addition, HP1%, HP2%—model chicken products with 1 or 2% of honey powder addition.

The product’s color is one of the key quality parameters that consumers have in mind when selecting it. Adding honey powder caused a notably more red color, and visible brown areas on the crust of chicken products after heat treatment (Figure 1).

The addition of honey powder did not differ significantly (*p* > 0.05) in the values of the L* parameter (lightness) of the products on any day of storage (Table 4). Model chicken products with honey powder (HP1% and HP2%) exhibited significantly (*p* ≤ 0.05) higher values of the a* color parameter and lower values of the b* color parameter than the control (C) product, regardless of the storage time.

During storage, an increase in values of the L* color parameter was noted in all variants of products. However, only in control (C) products were the differences significant (*p* ≤ 0.05). A significant (*p* ≤ 0.05) increase in L* parameter values was found between days 0 and 7, while no difference was found between days 7 and 14. The values of the a* color parameter also were observed to increase during storage, with significant (*p* ≤ 0.05) differences being found for the control and HP2% products. A significant (*p* ≤ 0.05) increase in a* parameter values was noticed between days 0 and 7, while there was no difference between days 7 and 14. Regardless of the variant of the model chicken product, the measured values of the b* color parameter were unaffected by storage time.

The total color difference (ΔE) values indicated the color difference between the control model chicken product and products with honey powder addition. The ΔE values shown in Table 4 were greater than 2 for each storage time, suggesting that color differences could be perceived by an inexperienced observer [12].

Additives used in recipes can affect the color of meat products in diverse ways. This depends not only on the amount of additive used but also on its properties. For example, the addition of turmeric to baked chicken meatballs [3] or propolis extract to poultry sausages [11] decreased the L* parameter values and increased the b* parameter values in the analyzed products. A similar trend was demonstrated by Turhan et al. [33] when bee pollen was added to beef meatballs.

Thermal processing changes the color of meat products due to protein denaturation and loss of water and fat [42,43]. Color change strongly depends on the cooking technique (boiling, roasting, frying, grilling) and the conditions under which it is carried out—temperature, time, or environment/medium [44,45]. In addition, non-enzymatic browning processes can occur during selected thermal processing methods such as roasting or grilling, which include reactions of denatured proteins with reducing sugars (Maillard reactions), the phenomenon of caramelization of sugars, as well as a number of other related reactions [45,46]. In addition, the naturally present sugars in meat and honey powder contributed to the creation of melanoidins—colorful products formed during the Maillard reaction [46]. Melanoidins were responsible for the darker and more red color of the HP1% and HP2% products compared to the control (C) products (Figure 1, Table 4). The darker and more red color of the products with the honey powder addition could also be due to the caramelization of the sugars present in the honey powder [19].

### 3.5. Lipid Oxidation of Model Chicken Products

Lipid oxidation can lead to deterioration in nutritional value, flavor, texture, and appearance of foods, reduce the shelf-life, and result in economic losses [47]. The addition of honey powder to chicken products significantly (*p* ≤ 0.05) reduced the lipid oxidation process (Table 5). After production, the malondialdehyde (MDA) content in model chicken products was low in all studied variants (0.37–0.39 mg MDA/kg of product). TBARS values significantly (*p* ≤ 0.05) increased during storage. The control products (C) were more susceptible to oxidation (0.66 mg MDA/kg after 7 days of storage and 1.17 mg MDA/kg after a 14-day period of storage) than HP products (0.99 and 0.60 mg MDA/kg for HP1% and HP2%, respectively, after 14 days of storage). On days 7 and 14, a significant (*p* ≤ 0.05) difference in TBARS value between HP1% and HP2% products was noted, with significantly lower values observed for HP2% products. This means that the lipid oxidation was slowest in products with a higher addition of honey powder. Adding honey powder to turkey breast can reduce unfavorable changes in lipids [15,16], which was explained by authors by the presence of reducing sugars in honey. Other studies have shown that adding bee pollen to beef meatballs can decelerate lipid oxidation [33].

The obtained results of TBARS values indicate that the addition of honey powder limited unfavorable changes in the fat of model chicken products stored under refrigerated conditions for 7 and 14 days. From a study performed by Zhang et al. [48], TBARS values in the range of 2.0–2.5 mg MDA/kg indicate the accepted limit at which meat and meat products do not go rancid.

Lipid oxidation can also be assessed using the Pressure Differential Scanning Calorimetry (PDSC) method. The fat extracted from the model chicken products with honey powder (HP1% and HP2%) was characterized by greater oxidative stability than the fat extracted from the control product (C), as evidenced by a significantly (*p* ≤ 0.05) longer oxidation induction time (OIT) after a 7- and 14-day period of storage (Table 5). Moreover, the control product exhibited a significantly (*p* ≤ 0.05) shorter OIT already after a 7-day period of storage, while for the HP2% product, no changes in OIT values during 14 days of storage were observed. Furthermore, fat extracted from the HP2% product had a significantly (*p* ≤ 0.05) longer OIT compared to the fat from the HP1% product on days 7 and 14 of storage, which means that the higher amount of honey powder provides the greater antioxidant effect. The results of OIT of the fat extracted from the model chicken products are consistent with the results of the TBARS values. For products with the addition of honey powder, lower TBARS values were observed, corresponding to a longer oxidation induction time, indicating effective inhibition of lipid oxidation and the potential use of this additive in producing such types of meat products.

The inhibitory influence on the lipid oxidation process that occurred in model chicken products with honey powder added could be linked to the biologically active compounds naturally present in honey, such as polyphenols, tocopherols, organic acids, vitamin C, enzymes, and peptides [20,24]. Furthermore, Maillard reaction products (MRPs) have also been demonstrated in the literature to prevent lipid oxidation in meat products such as ground chicken breast [49] or ground turkey breast [50].

### 3.6. Microbiological Quality of Model Chicken Products

Microbiological quality is an important factor, which informs about the safety and suitability for consumption of meat products. The microbiological quality of model chicken products is shown in Table 6. No Enterobacteriaceae were detected in any of the analyzed products for all storage times. Moreover, after a 14-day storage period of the products, the maximum limit of 5 log CFU/g for total mesophilic count (TMC), psychrotrophic bacteria, and lactic acid bacteria (LAB) for meat products was not exceeded [51]. Therefore, it can be concluded that the tested model chicken products in the proposed storage model met the microbiological quality criteria. It was observed that the addition of honey powder significantly (*p* ≤ 0.05) affected the count of psychrotrophic bacteria and LAB after 14 days of storage. The number of these bacteria was significantly (*p* ≤ 0.05) lower for HP1% and HP2% products than for the control product. The storage time of model chicken products also influenced the microbiological quality. In control (C) products, the number of mesophilic and psychrotrophic bacteria was higher after just 7 days, while for HP2% products only after 14 days of storage.

According to Fyfe et al. [52], not all honeys are equally effective at preventing the growth of diverse microorganisms due to a different composition of polyphenolic compounds and other substances. Additionally, some bacteria are more susceptible to the antimicrobial effects of honey, which was also proved in the current study. Osés et al. [20] showed that the biological properties of honey powder depended primarily on the type of carrier used during the drying process. The antimicrobial activities of honey powders against *Escherichia coli, Listeria monocytogenes,* and *Staphylococcus aureus* were lower than that of raw honey.

### 3.7. Sensory Quality of Model Chicken Products

As consumers make food choices in the marketplace, studying factors influencing sensory quality is critical to food development. The evaluated attributes of model chicken products included color, odor, flavor and taste cues, texture, and overall sensory quality.

**Figure 2:**
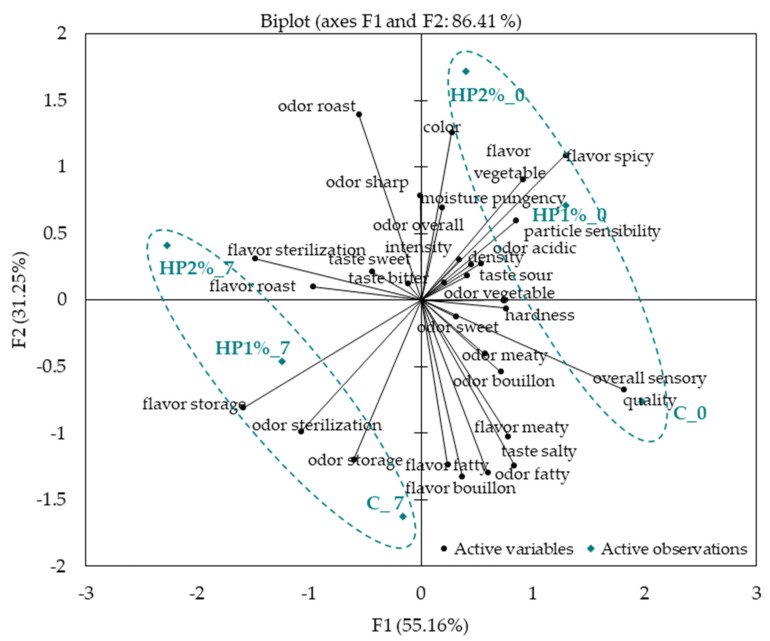
Principal Component Analysis plot of the similarities and differences in the sensory profiling of model chicken products. C—control model chicken product—without honey powder addition, HP1%, HP2%—model chicken products with 1 or 2% of honey powder addition; 0—without storage, 7—7 days of storage.

The PCA differences and similarities in the sensory profile of model chicken products during refrigerated storage are presented in Figure 2. It was found that the first principal component (PC1) accounted for 55.16% of the variability, whilst the second principal component (PC2) accounted for 31.25% of the variability. The most important determinants of the first principal component were vegetable, sterilization, and overall intensity odors; hardness, sterilization, storage, and roast flavors; and overall sensory quality. The second principal component was primarily determined by color, fatty, roast, storage, and sharp odors, as well as bouillon and fatty flavors.

A clear differentiation of model chicken products at the beginning and after 7 days of storage can be observed in the PCA map. The products tested at the beginning of storage (C, HP1% and HP2%) formed one cluster. The control (C) product was closely situated to overall sensory quality and attributes such as meaty odor and flavor, bouillon odor, salty taste, sweet odor, and hardness. The products with the addition of honey powder (HP1% and HP2%), were located at some distance from the control product, close to vegetable and spicy flavor, pungency, color, moisture, and overall odor intensity. On the other hand, the products stored for 7 days were found to be close to storage odor and flavor, and sterilization odor and flavor, which contributed to their distinct sensory characteristics, compared to products that were not stored.

**Figure 3:**
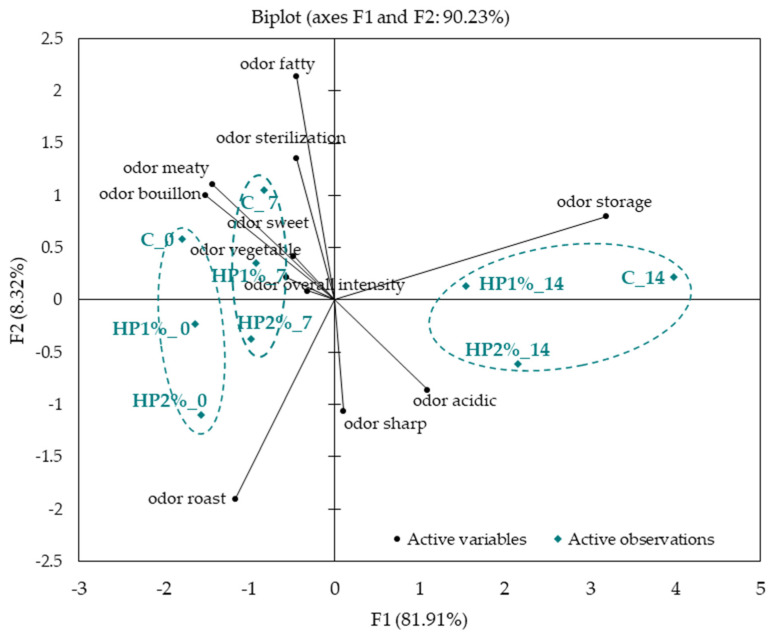
Principal Component Analysis plot of the similarities and differences in odor characteristics of model chicken products. C—control model chicken product—without honey powder addition, HP1%, HP2%—model chicken products with 1 or 2% of honey powder addition; 0—without storage, 7—7 days of storage, 14—14 days of storage.

On day 14 of storage, model chicken products were evaluated only for odor (excluding oral evaluation) due to the negative sensory perception. The differences and similarities in the odor profile of model chicken products without storage and stored for 7 and 14 days are presented in Figure 3. A clear difference was noted in the odor characteristics between model chicken products evaluated in each storage time. All variants of products (C, HP1% and HP2%) stored for 14 days were located in a cluster close to storage and acidic odor. Noticeably smaller differences were found in the sensory characteristics between chicken products on days 0 and 7, forming the remaining two separate clusters.

The available literature examined the addition of bee products as natural antioxidants to meat products, considering factors such as microbiological stability, lipid oxidation, storage conditions, and sensory quality [53]. As far as we are aware, no information can be found in the literature on the detailed sensory profiling of such meat products as chicken products containing honey powder, taking into account storage conditions. According to Reis et al. [54], the sensory profile of burger meat with microencapsulated propolis was characterized by lower grades in the odor and flavor but similar grades for color, appearance, and texture such as the control burger meat. Similarly, our study revealed that the honey powder addition also affected the sensory characteristics of model chicken products, providing less linkage to attributes such as fatty, bouillon, and meaty flavors and odors, salty taste, and overall sensory quality.

### 3.8. Volatile Compounds Profile of Model Chicken Products

The profile of volatile organic compounds (VOCs) creating the flavor of meat products is determined by many factors associated among others with product ingredients, parameters of technological process, as well as duration and conditions of storage. It is developed from non-volatile precursors as a result of lipid oxidation, Maillard reactions, interactions between the products of Maillard reaction and lipid oxidation, and microorganism metabolism [55,56].

**Figure 4:**
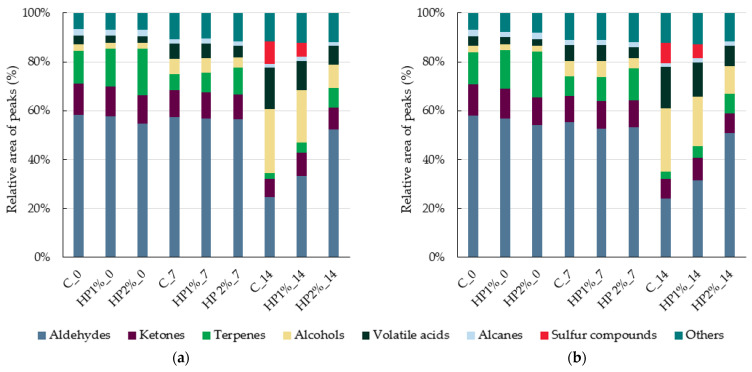
Profile chart of the main volatile compounds for the MXT-5-FID1 (**a**) and MXT-1701-FID2 (**b**) column identified in the headspace of samples of model chicken products. C—control model chicken product—without honey powder addition, HP1%, HP2%—model chicken products with 1 or 2% of honey powder addition; 0—without storage, 7—7 days of storage, 14—14 days of storage.

The profile charts of the VOCs recognized by e-nose in model chicken products are presented in Figure 4, and the main volatile compounds are presented in Table S1. The model chicken products (C, HP1%, and HP2%) just after production (day 0) demonstrated a similar profile of VOCs. It was found that the dominant detected volatile compounds were aldehydes (Figure 4), mainly hexanal (Table S1). Hexanal is an important volatile compound of cooked poultry meat derived from fatty acids’ oxidation process (mainly α-linolenic acid) occurring during thermal processes [57,58]. Moreover, the relative area of aldehydes for all chicken products at day 0 was at a comparable level. This was consistent with the TBARS results, which were also at a comparable level at the beginning of storage time (0.37–0.39 mg MDA/kg) for all tested products (Table 5).

Besides aldehydes, ketones and terpenes were also identified (Figure 4). The 2,3-pentanedione with the characteristic butter, creamy, burnt, and sweet odors was dominant among ketones (Table S1). Greater diversity was observed among terpenes, as 1,8-cineole, beta-pinene, alpha-phellandrene, limonene, and myrcene were identified in all samples (Table S1). The relative area of terpenes in the tested chicken products, regardless of storage time, was higher in products with honey powder addition in comparison to control (C) products due to terpenes commonly occurring in honey [59].

After a 7-day period of storage, minor changes in the profile of volatile compounds were noted. The proportion of aldehydes was comparable to the proportion found on day 0. A slight increase in the proportion of alcohols and volatile acids was observed; at the same time, the relative peak area of terpenes slightly decreased (Figure 4). More visible changes occurred after 14 days of storage. For control (C) and HP1% products, a high increase in the proportion of alcohols was observed (Figure 4), mainly identified as ethanol and 2-butanol (Table S1). Among the volatile acids, the proportion of acetic acid and pentanoic acid increased in all products. Moreover, 2-methylbutanoic acid and propanoic acid were detected in all tested products (Table S1). After a 14-day storage period, for the control (C) and HP1% products the proportion of aldehydes decreased; at the same time, the sulfur compounds (dimethyl disulfide and 2-mercaptoethanol) were detected. Additionally, in the control (C) product, these changes were more notable than in HP1% products (relative area of peaks observed for alcohols and sulfide compounds were higher). In products with a 2% of honey powder addition (HP2%), an increase in alcohols and volatile acids was mainly observed, while dimethyl disulfide and 2-mercaptoethanol were not identified. Differentiation in the proportion of volatile compound groups is linked to the fact that hexanal is formed in the initial phases of oxidation and may undergo further reactions, which lead to a reduction in its content [60]. Further reactions and progressive oxidation can produce alcohols or sulfur compounds [61]. This phenomenon is confirmed by the results of TBARS after 14 days of storage, as higher values were obtained for control (C) and HP1% products than for HP2% products (Table 5), indicating greater oxidative stability of HP2% products. Furthermore, increased content of alcohols may inform about the metabolism of glucose and amino acids by microorganisms [62]. The counts of tested bacteria were significantly higher in the control (C) and HP1% products after 14 days compared to day 0 (Table 6).

The above-mentioned changes and found compounds may explain the unfavorable changes in the sensory odor profile of the products after 14 days of storage (Figure 3). Volatile compounds formed during the storage of meat products are mainly the result of lipid oxidation and microbial metabolism of glucose and amino acids [62]. Chicken meat is more susceptible to flavor deterioration than red meat because it contains higher levels of unsaturated fatty acids [6,63]. According to Mikš-Krajnik et al. [51], spoilage in chicken meat is a two-stage process. Initially, an increase in the level of free fatty acids and alcohols is observed (primary spoilage), and then there is an increase in the sulfur compounds amount (secondary spoilage). In addition, due to the degradation of sulfur-containing amino acids (cysteine and methionine), dimethyl disulfide in tested chicken products may be identified [64]. Therefore, except hexanal, ethanol and dimethyl disulfide can be considered a chemical marker of spoilage in chicken meat [65]. Based on the results obtained in our study, it can be stated that adding 2% of honey powder slowed down the unfavorable lipid oxidation, compounds degradation, and other reactions occurring in model poultry products during storage.

**Figure 5:**
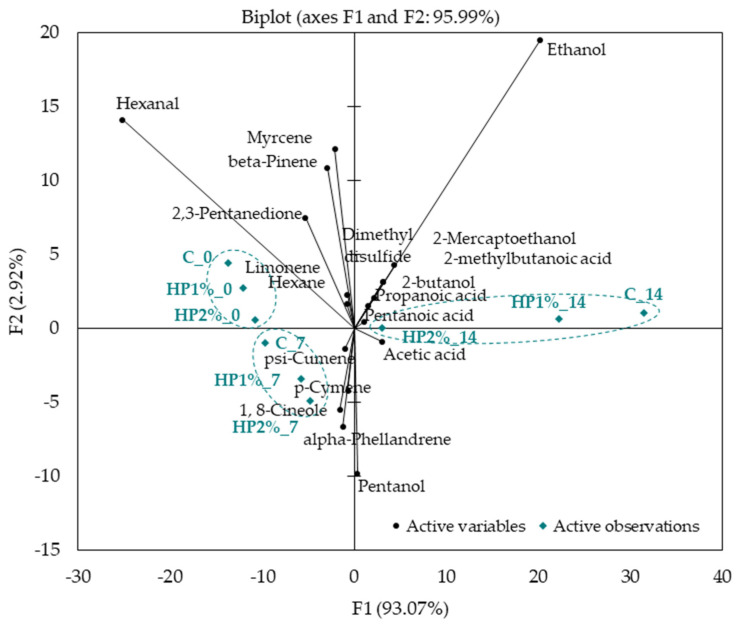
Principal Component Analysis plot of the similarities and differences in the profile of the main volatile compounds identified in the headspace of model chicken products. C—control model chicken product—without honey powder addition, HP1%, HP2%—model chicken products with 1 or 2% of honey powder addition; 0—without storage, 7—7 days of storage, 14—14 days of storage.

The Principal Component Analysis (PCA) was carried out to determine the similarities and differences in the profile of the VOCs recognized by e-nose in model chicken products (Figure 5). The first two principal components (PC1 and PC2) accounted for 95.99% of the total variance, whereas PC1 accounted for the majority (93.07%) of the total variance. The main volatile compound differentiating chicken products (C, HP1%, and HP2%) without storage (day 0) was hexanal. It was found that all products after the 7 days of storage were located close to such volatile compounds as p-cymene and 1,8-cineole (in the III quadrant). However, control (C) and HP1% products after 14 days of storage were located on the opposite side (in the I quadrant) at a large distance from other samples. Those products represented a different profile of VOCs, showing the presence of ethanol and sulfur compounds. On day 14 of storage, the product with 2% honey powder addition (HP2%) was located near the center of the coordinate system, at some distance from the control (C) and HP1% products.

## 4. Conclusions

Adding the honey powder (1 and 2%) improves the quality characteristics of the model chicken products. The honey powder was beneficial in inhibiting the lipid oxidation process during a 14-day period of refrigerated storage, revealed by reduced TBARS index values and longer oxidation induction time compared to the control product. Moreover, lipid oxidation was slower during storage in HP2% than in HP1% chicken products. The addition of honey powder also reduced the growth of psychrotrophic bacteria and LAB stored for 7 and 14 days compared to the control chicken product.

Honey powder addition altered the sensory characteristics of model chicken products compared to the control ones; however, more remarkable changes in sensory characteristics were observed during the storage. The use of honey powder did not limit the formation of unfavorable flavors (storage and sterilization) after 7 days and unfavorable odors (storage and acidic) after 14 days of storage. Moreover, after a 14-day period of storage, model chicken products were characterized by different volatile compound profiles, with a higher proportion of volatile acids and alcohols, compared to the products stored for 0 and 7 days. Furthermore, sulfur compounds were detected in C and HP1% chicken products, indicating sulfur-containing amino acids (cysteine and methionine) degradation.

Honey powder appears to be an emerging promising natural compound that can extend the shelf-life of model chicken products stored under refrigerated conditions. Nevertheless, some modifications in the recipe, like using more intense herbs and spices, could be helpful to improve the sensory desirability of chicken products.

## Figures and Tables

**Table 1:** Cooking loss and basic chemical composition of model chicken products (average values ± standard deviation).

	C	HP1%	HP2%
Cooking loss (%)	9.1 ± 0.5 ^a1^	8.7 ± 0.7 ^a^	8.7 ± 0.6 ^a^
Water (%)	69.5 ± 0.2 ^b^	68.1 ± 0.3 ^a^	67.4 ± 0.5 ^a^
Protein (%)	19.3 ± 0.4 ^b^	19.1 ± 0.1 ^ab^	18.6 ± 0.2 ^a^
Fat (%)	6.8 ± 0.3 ^a^	7.0 ± 0.3 ^a^	7.1 ± 0.4 ^a^
Carbohydrates (%)	1.3 ± 0.1 ^a^	2.8 ± 0.2 ^b^	4.0 ± 0.2 ^c^
Salt (%)	1.9 ± 0.2 ^a^	1.8 ± 0.1 ^a^	1.8 ± 0.1 ^a^

C—control model chicken product—without honey powder addition, HP1%, HP2%—model chicken products with 1 or 2% of honey powder addition. ^1^ Average values with the same letter within a row (^a,b,c^) are not significantly different (Tukey’s HSD test, *p* ≤ 0.05).

**Table 2:** Changes in the water activity of model chicken products during refrigerated storage (average values ± standard deviation).

Days of Storage	C	HP1%	HP2%
0	0.975 ± 0.003 ^ax1^	0.975 ± 0.002 ^ax^	0.975 ± 0.002 ^ax^
7	0.973 ± 0.001 ^ax^	0.977 ± 0.001 ^ax^	0.973 ± 0.004 ^ax^
14	0.976 ± 0.001 ^ax^	0.975 ± 0.002 ^ax^	0.975 ± 0.002 ^ax^

C—control model chicken product—without honey powder addition, HP1%, HP2%—model chicken products with 1 or 2% of honey powder addition. ^1^ Average values with the same letter within a row (^a^) and a column (^x^) are not significantly different (Tukey’s HSD test, *p* ≤ 0.05).

**Table 3:** Changes in the shear force (N) of model chicken products during refrigerated storage (average values ± standard deviation).

Days of Storage	C	HP1%	HP2%
0	14.29 ± 1.10 ^ax1^	14.57 ± 1.43 ^ax^	14.95 ± 1.23 ^ax^
7	14.48 ± 1.16 ^axy^	14.13 ± 1.35 ^ax^	15.19 ± 0.94 ^ax^
14	15.55 ± 1.33 ^ay^	14.52 ± 1.49 ^ax^	14.34 ± 1.14 ^ax^

C—control model chicken product—without honey powder addition, HP1%, HP2%—model chicken products with 1 or 2% of honey powder addition. ^1^ Average values with the same letter within a row (^a^) and a column (^x,y^) are not significantly different (Tukey’s HSD test, *p* ≤ 0.05).

**Table 4:** Changes in the color parameters of model chicken products during refrigerated storage (average values ± standard deviation).

Days of Storage	C	HP1%	HP2%
	L* (─)		
1	67.23 ± 1.81 ^ax1^	67.88 ± 1.69 ^ax^	67.25 ± 1.83 ^ax^
7	69.30 ± 1.28 ^ay^	69.13 ± 1.98 ^ax^	68.06 ± 1.74 ^ax^
14	69.19 ± 1.84 ^ay^	69.11 ± 1.70 ^ax^	67.88 ± 1.14 ^ax^
	a* (─)		
1	2.79 ± 0.59 ^ax^	4.85 ± 0.85 ^bx^	4.71 ± 0.95 ^bx^
7	3.39 ± 0.55 ^ay^	5.29 ± 0.95 ^bx^	5.61 ± 0.68 ^by^
14	3.67 ± 0.69 ^ay^	5.38 ± 0.68 ^bx^	6.20 ± 0.77 ^cy^
	b* (─)		
1	14.97 ± 0.76 ^bx^	13.35 ± 1.15 ^ax^	13.47 ± 1.95 ^ax^
7	15.43 ± 1.20 ^bx^	14.27 ± 1.26 ^ax^	13.58 ± 0.94 ^ax^
14	15.51 ± 0.86 ^bx^	13.80 ± 0.71 ^ax^	13.47 ± 1.18 ^ax^
	ΔE (─)		
1	–	3.16 ± 1.41 ^a^	3.22 ± 1.07 ^a^
7	–	3.89 ± 0.98 ^a^	3.76 ± 0.74 ^a^
14	–	2.94 ± 0.93 ^a^	3.82 ± 0.93 ^b^

C—control model chicken product—without honey powder addition, HP1%, HP2%—model chicken products with 1 or 2% of honey powder addition. ^1^ Average values with the same letter within a row (^a,b,c^) and a column (^x,y^) are not significantly different (Tukey’s HSD test, *p* ≤ 0.05).

**Table 5:** Changes in the TBARS values and oxidation induction time (OIT) of model chicken products during refrigerated storage (average values ± standard deviation).

Days of Storage	C	HP1%	HP2%
	TBARS (mg MDA/kg)		
1	0.37 ± 0.04 ^ax1^	0.38 ± 0.02 ^ax^	0.39 ± 0.03 ^ax^
7	0.66 ± 0.02 ^cy^	0.50 ± 0.02 ^by^	0.42 ± 0.05 ^ax^
14	1.17 ± 0.05 ^cz^	0.99 ± 0.03 ^bz^	0.60 ± 0.02 ^ay^
	OIT (min)		
1	16.93 ± 0.75 ^az^	16.80 ± 1.18 ^ax^	17.78 ± 0.40 ^ax^
7	13.86 ± 0.49 ^ay^	16.67 ± 0.13 ^bx^	17.61 ± 0.32 ^cx^
14	12.47 ± 0.11 ^ax^	15.69 ± 0.51 ^bx^	17.62 ± 0.30 ^cx^

C—control model chicken product—without honey powder addition, HP1%, HP2%—model chicken products with 1 or 2% of honey powder addition. ^1^ Average values with the same letter within a row (^a,b,c^) and a column (^x,y,z^) are not significantly different (Tukey’s HSD test, *p* ≤ 0.05).

**Table 6:** Changes in the microbiological quality (log CFU/g) of model chicken products during refrigerated storage (average values ± standard deviation).

Days of Storage	C	HP1%	HP2%
	Enterobacteriaceae		
1	nd	nd	nd
7	nd	nd	nd
14	nd	nd	nd
	Total Mesophilic Count (TMC)		
1	2.15 ± 0.23 ^ax1^	2.18 ± 0.13 ^ax^	2.35 ± 0.16 ^ax^
7	2.62 ± 0.03 ^by^	1.91 ± 0.12 ^ay^	2.07 ± 0.24 ^ax^
14	2.98 ± 0.12 ^ay^	3.20 ± 0.03 ^az^	2.90 ± 0.19 ^ay^
	Psychrotrophic Bacteria		
1	1.26 ± 0.45 ^ax^	1.20 ± 0.17 ^ax^	1.52 ± 0.07 ^ax^
7	2.83 ± 0.10 ^by^	2.24 ± 0.18 ^by^	1.37 ± 0.21 ^ax^
14	2.92 ± 0.19 ^by^	2.05 ± 0.02 ^ay^	1.74 ± 0.13 ^ay^
	Lactic Acid Bacteria (LAB)		
1	1.56 ± 0.24 ^ax^	1.30 ± 0.30 ^ax^	1.46 ± 0.15 ^ax^
7	2.66 ± 0.08 ^by^	1.25 ± 0.18 ^ax^	1.52 ± 0.24 ^ax^
14	2.23 ± 0.16 ^by^	1.42 ± 0.10 ^ax^	1.33 ± 0.35 ^ax^

C—control model chicken product—without honey powder addition, HP1%, HP2%—model chicken products with 1 or 2% of honey powder addition. nd—not detectable. ^1^ Average values with the same letter within a row (^a,b^) and a column (^x,y,z^) are not significantly different (Tukey’s HSD test, *p* ≤ 0.05).

## Data Availability

The original contributions presented in the study are included in the article/Supplementary Material, further inquiries can be directed to the corresponding authors.

## Supplementary Materials

The following supporting information can be downloaded at: https://www.mdpi.com/article/10.3390/foods13244163/s1, Table S1: Volatile compounds recognized by AroChemBase in model chicken products (e-nose analysis).
