# Supplementary material for: The Effect of Honey Powder Addition on Chosen Quality Properties of Model Chicken Products
Source: Foods. 2024 Dec 22;13(24):4163. doi: 10.3390/foods13244163 (PMC11675915; doi:10.3390/foods13244163)
Supplement: Supplementary file 1 [file foods-13-04163-s001.zip › foods-3345681-supplementary.pdf]

Table S1. Volatile compounds recognized by AroChemBase in model chicken products (e-nose analysis).

Possible Matches Compounds[1]	Sensory Descriptors[1]	KI MXT-5 *	KI MXT-1701 **	Days of Storage								
				0			7			14		
				C	HP1%	HP2%	C	HP1%	HP2%	C	HP1%	HP2%
**Aldehydes**												
Hexanal	fatty, green, fresh, herbaceous, fruity	801	895	x	x	x	x	x	x	x	x	x
**Ketones**												
2,3-Pentanedione	butter, creamy, sweet, burnt	689	789	x	x	x	x	x	x	x	x	x
**Terpenes**												
1,8-Cineole	herbaceous, licorice, pine, sweet, camphor	1043	1086	x	x	x	x	x	x	x	x	x
beta-Pinene	dry, green, pine, resinous, sweet	984	998	x	x	x	x	x	x	x	x	x
alpha-Phellandrene	citrus, green, spicy, terpenic, woody	1006	1019	x	x	x	x	x	x	x	x	x
Limonene	citrus, fruity, orange	1045	1073	x	x	x	x	x	x	x	x	x
Myrcene	balsamic, etheral, fruity, lemon,	996	1020	x	x	x	x	x	x	x	x	x
**Alcohols**												
Pentanol	alcoholic, balsamic, fruity, mild, sweet	770	881	x	x	x	x	x	x	x	x	x
Ethanol	alcoholic, pungent, strong	463	560							x	x	x
2-butanol	strong, wine	598	696							x	x	
**Volatile Acids**												
Acetic acid	acetic, acidic, pungent	625	800	x	x	x	x	x	x	x	x	x
Pentanoic acid	acidic, cheese, pungent,	903	1077	x	x	x	x	x	x	x	x	x
2-methylbutanoic acid	cheese, fruit (overripe), pungent	873	1018							x	x	x
Propanoic acid	acidic, pungent, rancid	712	882							x	x	x
**Alcanes**												
Hexane		596	625	x	x	x	x	x	x	x	x	x
**Sulfur Compounds**												
Dimethyl disulphide	cabbage, garlic, onion, sulfurous	737	800							x	x	
2-Mercaptoethanol	strong, sulfurous	552	894							x	x	
**Others**												
psi-Cumene	aromatic, herbaceous	994	1047	x	x	x	x	x	x	x	x	x
p-Cymene	aromatic, balsamic, fruity, lemon, mild	1026	1072	x	x	x	x	x	x	x	x	x

C – control model chicken product – without honey powder addition, HP1%, HP2% – model chicken products with 1 or 2% of honey powder addition.

[1] volatile compound recognized and the sensory descriptions for the listed compounds are taken from AroChemBase; * Kovats index for non-polar column; ** Kovats index for slightly polar column.
